# Carbolic Acid Preparations

**Published:** 1870-11

**Authors:** 


### Carbolic Acid Preparations.

Mr. T. A. Baldwin, in a paper read before the British Pharmaceutical Conference, gives the following as advisable proportions in the use of carbolic acid:

As a rule, it is better to dissolve the crystallized carbolic acid in the proportion of one part by weight of the acid to six of glycerine (carbolate of glycerine). In this state it can be diluted equally indefinitely.

In general, a dose of carbolic acid is 1 grain in an ounce of water.

As a gargle, 1 or 2 grains to an ounce of water.

As an injection 1 grain to 4 ounces of water.

As a lotion, 15 grains to an ounce of water.

As an ointment, 30 grains to an ounce of benzoated lard.

As a liniment, 1 grain to 20 ounces of olive oil.

As a plaster, 1 part of carbolic acid to 3 of shellac.

The crystallized carbolic acid to be used as a caustic.

The carbolate of glycerine, as above should be used in 1 or 2 drop doses.

Antiseptic oil for abcesses, 1 part of acid to 4 of boiled linseed oil.

Antiseptic putty, 6 spoonsful of the antiseptic oil mixed with common whiting.

Aqueous solution of carbolic acid is 1 part of acid to 40 of water (1 ounce of acid to a quart of hot water, well agitated and filtered.)

Sick rooms to disinfect: place a portion of the dissolved acid in a porcelain dish, and float it in a larger vessel of hot water.

Disinfecting purposes generally: 1 pound of *crystals* to 6 gallons of water. *Fluid,* 1 part to 80 of water. *Powder,* 1 ounce of crystals with 4 pounds of slaked lime.

For drains: take 1 pound of the fluid carbolic acid to 5 gallons of warm water.

Toothache is often cured with 1 drop of carbolate of glycerine; and diarrhœa arrested in half an hour with 2 drops.

In all cases of parasitic life, it is advisable to commence with very dilute carbolate of glycerine.—[*Chemist and Druggist—Drug Circ.*

